# Sexual dimorphism in the Chinese endemic species *Pachyhynobius shangchengensis* Fei, Qu and Wu, 1983 (Urodela: Hynobiidae)

**DOI:** 10.7717/peerj.6408

**Published:** 2019-02-19

**Authors:** Jianli Xiong, Baowei Zhang, Qiangqiang Liu, Tao Pan, Jianping Gou

**Affiliations:** 1 College of Animal Science and Technology, Henan University of Science and Technology, Luoyang, Henan, China; 2 School of life Science, Anhui University, Hefei, Anhui, China

**Keywords:** Sexual shape dimorphism, Sexual selection, Morphometrics, Shangcheng stout salamander, Sexual size dimorphism

## Abstract

Sexual dimorphism (SD) is a widespread phenomenon in most vertebrate species and is exhibited in a myriad of ways. In amphibians, sexual size dimorphism, in which females are larger than males, is the most common type, and sexual shape dimorphism varies among species. Different selection forces (sexual selection, fecundity selection, and ecological selection) that act differently upon the sexes form the consequence of SD. Thus, studies of SD provide information about the general intersexual divergence of the same species and allow insights into the impact of selective forces on the sexes. In this study, we analyzed morphometric data of the Shangcheng stout salamander, *Pachyhynobius shangchengensis*, an endemic and poorly known Chinese salamander, to examine sexual dimorphism in size and shape. The morphometric data included 15 characteristics of 68 females and 55 males which were analyzed using univariate and multivariate methods. A significant difference was found between the sexes in terms of both body size (snout-vent length) and some body shapes (e.g., head length and width, tail length and width, distance between limbs, and limb length and width) in this salamander. The longer snout-vent length in males may be attributed to sexual selection, longer and wider head in males may contribute to male-male competition, longer and wider tail in males may be attributed to energy storage and reproductive success, the larger distance between limbs in females is likely due to a fecundity advantage, and longer and more robust limbs in males may be related to reproductive or competitive behaviors. These results demonstrated that sexual dimorphism of different morphological traits is the consequence of different selection forces that act differently upon the sexes.

## Introduction

Sexual dimorphism (SD) refers to differences in traits between males and females within the same species (Berns, 2013). Sexual dimorphism is a widespread phenomenon of varying degrees in most vertebrate species (Shine, 1979; Andersson, 1994; Kupfer, 2007) and is exhibited in a myriad of ways, e.g., coloration, ornamentation, behavior, body size and shape (Duellman & Trueb, 1994; Berns, 2013). In general, body size or mass SD of sexually mature organisms is called sexual size dimorphism (SSD), and dimorphism in other morphological characteristics is called sexual shape dimorphism (SShD). Females are larger than males is the most common type of SSD in amphibians (e.g., Shine, 1979; Ivanović et al., 2008; Romano, Bruni & Paoletti, 2009; Seglie, Roy & Giacoma, 2010; Amat et al., 2015; Reinhard & Kupfer, 2015; Altunışık, 2017). Sexual dimorphism is the consequence of different selection forces that act differently upon the sexes (Blanckenhorn, 2005). Sexual selection, fecundity selection, and ecological selection have been proposed as the main driving forces explaining the existence and evolution of SD (Shine, 1988; Hedrick & Temeles, 1989; Andersson, 1994; Jockusch, 1997; Blanckenhorn, 2005; Fontenot & Seigel Jr, 2008; Berns, 2013; Xiong et al., 2017). Studies on SD provide an excellent opportunity to examine the putative selective basis for divergence in morphological traits (Dubey, Chevalley & Shine, 2011).

Family Hynobiidae is the third largest family in the Order Urodela, with two subfamilies, 10 genera, and 68 species (Frost, 2018). Studies on SD of hynobiid salamanders include species in the genera *Hynobius* (e.g., Kakegawa et al., 2017; Xiong et al., 2017), *Liua* (Zhang et al., 2014), *Pseudohynobius* (Xiong et al., 2016a), *Salamandrella* (Hasumi, 2010; Xiong et al., 2017), and *Onychodactylus* (Xiong et al., 2016b). Sexual size dimorphism (male-biased) has been reported in *L. shihi* and *O. zhangyapingi*; head dimorphism (female-biased) was identified in *L. shihi*, *O. zhangyapingi*, and *S. keyserlingii*; limb dimorphism (male-biased) was reported in *L. shihi* and *H. leechii*; tail dimorphism (male-biased) was discovered in *O. zhangyapingi*, *S. keyserlingii* and *H. kimurae*; and body dimorphism (represented by trunk length, female-biased) has been detected in all reported hynobiids. Among these salamanders, *L. shihi* are aquatic throughout the year (permanently aquatic), the species of *Hynobius*, *S. keyserlingii*, *Ps. flavomaculatus* and *O. zhangyapingi* are mainly terrestrial, and aquatic only during breeding (Fei et al., 2006; Poyarkov et al., 2012). Sexual dimorphism of these reported hynobiid salamanders varies among different species, and no obvious pattern was found except female-biased trunk. This case was also found in other salamanders (e.g., Reinhard & Kupfer, 2015; Reinhard, Renner & Kupfer, 2015; Pogoda & Kupfer, 2018).

The Shangcheng stout salamander (*Pachyhynobius shangchengensis* Fei, Qu & Wu, 1983) is an endemic and poorly known Chinese salamander, which is only distributed in the Dabie Mountains of Southeastern China, including Huoshan, Yuexi, Jinzhai, Yingshan, and Shangcheng Counties of China (Fei et al., 2006).This salamander is an aquatic year-round species (permanently aquatic) that inhabits small hill streams with slow to moderate flowing water (Chen, 1992). The egg sacs have only been found in April and May in captivity, which containing 18–32 eggs with an average diameter of 3.3 mm, and metamorphosis occurred between 441 and 454 d after hatching at an average total length of 94.8 mm (Pasmans et al., 2012). Eye size showed no sexual dimorphism (Lv et al., 2014), but head width showed male-biased (Pasmans et al., 2012). Furthermore, dentition showed a noticeable sexual dimorphism, which males possesses pedicellate teeth with a chisel- or spearhead-like crown in the upper and lower jaw, but females have flattened teeth with small bladed labial and large bladed lingual cusps (Clemen & Greven, 2009). In this study, we explored sexual size and shape dimorphism in *Pa. shangchengensis* based on specimens collected from the field. Since sexual selection promotes morphological features which allow an individual to gain reproductive success (Andersson, 1994); fecundity selection favors morphological characters which improve reproductive output (Hedrick & Temeles, 1989; Jockusch, 1997); ecological selection favors morphological characteristics which maximize survival and growth (Shine, 1989; Fontenot & Seigel Jr, 2008). Furthermore, the aquatic hynobiid salamander, *L. shihi*, shows male-biased SSD and limb, and female-biased head (Zhang et al., 2014), as well as all reported hynobiid salamanders shows female-biased trunk and male-biased hindlimb length (e.g., Zhang et al., 2014; Xiong et al., 2016a; Xiong et al., 2016b; Xiong et al., 2017). We hypothesize that the aquatic *Pa. shangchengensis* shows male-biased SSD, male-biased characters are expected in proportions of the limbs, and female-biased traits are expected in head dimensions and trunk measurements.

## Material and Methods

### Sampling and data collection

A total of 123 (55 males and 68 females) adult *Pa. shangchengensis* were used in this study. These salamanders were caught by hand in the nighttime from the streams of Dabie Mountain, Anhui Province, China (30° 58′N, 116°04′E; 1,135 m above of sea level) in June 15, 2015. The breeding season may be in April and May according to the breeding in captivity (Pasmans et al., 2012). Thus, the specimens collected in this day may represent individuals in post-breeding or non-breeding season. Upon arrival at the laboratory, animals were euthanized via submergence in a buffered MS-222 solution and then stored in 10% formalin. Specimens were sexed by inspection of the gonads through a small ventro-lateral incision, and sexually mature was determined according to the development of gonads. To quantify intersexual differences in body size and shape, 15 variables were measured with digital calipers to the nearest 0.1 mm from the right side of each individual (Table 1), and each measurement was taken once.

**Table 1:** Definitions of the morphological character sets in *Pachyhynobius shangchengensis*.

Variables	Definition
snout-vent length	from the tip of snout to the posterior margin of the cloaca
head length	from the tip of the snout to the gular fold
head width	width of the head at its widest point
tail length	from the posterior margin of the cloaca to the tip of the tail
tail height	height of the tail at its highest point
tail width	width of the tail at its widest point (the maximum of cloaca)
eye diameter	maximum diameter of the eye on the horizontal axis
internarial space	space between nares
intercanthal space	minimum space between the anterior corners of the eyes
Length of forelimb	from the base of the forelimb to the tip of the longest finger
Length of hindlimb	from the base of the hindlimb to the tip of the longest toe
distance between limbs	distance between the posterior base of the forelimb (axilla) to the anterior base of the hindlimb (groin) on the same side
Cloaca length	maximum width of the cloaca
forelimb width	maximum width of the forelimb
hindlimb width	maximum width of the hindlimb

This research is in compliance with laws and ethical standards of China. All animal procedures were approved by the Animal Care and Use Committee of the College of Animal Science and Technology, Henan University of Science and Technology (CAST2015040010). All field work with animal was conducted according to relevant national and international guidelines.

### Data analysis

All characters measured were tested for normality using Kolmogorov–Smirnov test and homogeneity of variances (Levene’s test). Non-homogeneity variables were transformed using a LOG10 transformation. The sexual dimorphism of body size (SVL) was tested by one-way analysis of variance (ANOVA). Correlation of measured characters and body size (SVL) was carried out using the Pearson correlation analysis. Since all measured characters were highly correlated with body size (*P* < 0.001 in all cases), SShD was assessed by an analysis of covariance (ANCOVA) using SVL as a covariate to adjust the characters to head-body size, with SVL * sex as a second independent variable to test for differences in slope. When there was no difference in slope, the interaction term (SVL * sex) was dropped from the model and the analysis re-run. Sexual size dimorphism was calculated using the size dimorphism index (SDI) of Lovich & Gibbons (1992), in which SSD = (size of the larger sex/size of the smaller sex) +1, which is positive when females are the larger sex and negative when males are the larger sex. All statistical analysis was carried out with SPSS software, version 22.0 (SPSS Inc., Chicago, IL, USA). Values are presented as mean ± standard error of the mean, and the significance level was set to *α* = 0.05.

## Results

Table 2 presents the mean values and ranges of the original morphological measurements. Mean snout-vent length was greater in males (104.08 ± 1.13, *N* = 55) than in females (100.64 ± 1.00, *N* = 68). The results of ANOVA showed a significant difference in snout-vent length (*F*_1,121_ = 5.218, *P* = 0.024). Thus, males were longer than females, and the SDI was −2.04.

**Table 2:** Descriptive statistics of original morphometric characters (mm) in males and females of *Pachyhynobius shangchengensis*.

Variables	Female (*n* = 68)		Male (*n* = 55)	
	Mean ± S.E	Range	Mean ± S.E	Range
Snout-vent length	100.64 ± 1.00	84.76–120.18	104.08 ± 1.13	83.49–119.44
Head length	22.98 ± 0.26	18.77–27.92	24.49 ± 0.25	19.72–28.44
Head width	17.58 ± 0.21	13.58–21.32	19.35 ± 0.24	15.77–22.72
Tail length	65.12 ± 0.90	45.21–80.97	72.06 ± 1.02	54.69–88.18
Tail height	13.91 ± 0.25	10.01–18.78	14.47 ± 0.25	10.96–17.96
Tail width	12.33 ± 0.16	9.36–14.82	13.22 ± 0.19	10.45–16.11
Eye diameter	4.43 ± 0.04	3.62–5.28	4.47 ± 0.05	3.33–5.55
Internarial space	5.54 ± 0.06	4.27–6.64	5.74 ± 0.06	4.98–6.96
Intercanthal space	7.11 ± 0.07	5.46–8.34	7.55 ± 0.08	6.30–8.79
Length of forelimb	19.27 ± 0.17	15.86–22.36	20.79 ± 0.22	17.17–24.22
Length of hindlimb	23.58 ± 0.20	19.28–26.42	24.80 ± 0.23	19.83–28.10
Distance between limbs	48.89 ± 0.54	41.66–59.13	49.21 ± 0.65	39.66–57.74
Cloaca length	7.01 ± 0.12	4.30–10.13	7.27 ± 0.12	5.79–9.49
Forelimb width	3.61 ± 0.07	2.25–4.79	4.16 ± 0.09	2.56–5.25
Hindlimb width	6.64 ± 0.12	4.71–8.96	7.26 ± 0.13	4.91–9.25

**Figure 1:**
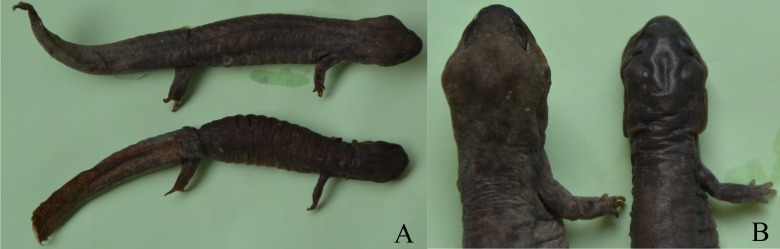
Sexual dimorphism in *Pachyhynobius shangchengensis*. (A) Overall view. Male (up) and female (down). (B) Sexual dimorphism of head and limbs. Male (left) and female (right). Left limbs were removed for other researches. These photos were taken by Tao Pan.

The ANCOVA results, which account for the effect of SVL, revealed that ten morphological variables (head length, head width, tail length, tail width, intercanthal space, length of forelimb, length of hindlimb, distance between limbs, forelimb width, and hindlimb width) were significantly different between the sexes (Table 3). Males had longer and wider head, longer and width tail, wider intercanthal space, longer and more robust forelimbs and hindlimb, but a shorter distance between limbs than females (Fig. 1).

**Table 3:** Analysis of sexual shape dimorphism in *Pachyhynobius shangchengensis*. Results of ANCOVA comparing 14 traits of sexes relatively to snout-vent lentgth (SVL).

Characters	*F*	*P*	Sex bias
Head length	15.351	<0.001	M
Head width	33.297	<0.001	M
Tail length	29.143	<0.001	M
Tail height	0.652	0.421	n.s
Tail width	8.199	0.005	M
Eye diameter	0.133	0.716	n.s
Internarial space	1.359	0.246	n.s
Intercanthal space	12.793	0.001	M
Length of forelimb	26.299	<0.001	M
Length of hindlimb	10.784	0.001	M
Distance between limbs	16.333	<0.001	F
Cloaca length	0127	0.723	n.s
Forelimb width	18.030	<0.001	M
Hindlimb width	8.519	0.004	M

**Notes.**

M male bias F female bias n.s no sex bias

## Discussion

Sexual size dimorphism is the most conspicuous sexual character in amphibian lineages (Shine, 1979; Andersson, 1994; Kupfer, 2007) and has important consequences for animal ecology and behavior (Kupfer, 2007). Three patterns of SSD have been described in mature amphibians according to body size: (1) females larger than males, female-biased SSD; (2) males larger than females, male-biased SSD; and (3) females equal in size to males, unbiased SSD (Shine, 1979; Bruce, 1993; Bruce, 2000). The first SSD pattern is the most common. Shine (1979) reported that females are larger in 60.8% of the 79 urodele species and in 89.6% of the 589 anurans. Sexual size dimorphism of *Pa. shangchengensis* was the second pattern, which has seldom been reported in salamander species, e.g., *Euprocuts platycephalus* (Bovero et al., 2003), *Phaeognathus hubrichti* (Bakkegard & Guyer, 2004), *L. shihi* (Zhang et al., 2014), and *O. zhangyapingi* (Xiong et al., 2016b). Although SSD is the result of different selection forces (sexual selection, fecundity selection, and natural selection) that equilibrate differently (Fairbairn, Blackenhorn & Szeke’ly, 2007; Colleoni et al., 2014), sexual selection is proposed to explain male-biased SSD (Monroe, South & Alonzo, 2015). Sexual selection favors larger males, which behave more aggressively with better fighting ability; thus increasing mating success (Shine, 1979; Bruce, 1993; Bakkegard & Guyer, 2004; Seglie, Roy & Giacoma, 2010). About 86.7% of urodele species in which males equal or exceed females in size engage in male combat (Shine, 1979). Aggressive behavior and male combat of *Pa. shangchengensis* has not been found in the field, but aggressive behavior (bite females) of males was witnessed on several occasions in captivity (Pasmans et al., 2012). Thus, aggressive behavior and male combat may be presented in male *Pa. shangchengensis*, and male-biased SSD of *Pa. shangchengensis* may be attributed to sexual selection.

Sexual dimorphism in head size (usually expressed in head length or head width or both) is found in many salamander species, in which males usually have a larger head than females (e.g., Bovero et al., 2003; Bakkegard & Guyer, 2004; Fontenot & Seigel Jr, 2008; Marvin, 2009; Hasumi, 2010; Zhang et al., 2014; Xiong et al., 2016b). Sexual, fecundity and ecological selection have been proposed to explain SD in head size (Zhang et al., 2014; Xiong et al., 2016b). Fecundity and ecological selection respond to female-biased head size, which favors females with larger heads that can consume more energy for reproductive investment and capture larger prey by avoiding competition for resources (Zhang et al., 2014; Xiong et al., 2017). Sexual selection is attributed to male-biased head size, which favors males with larger heads to become winners during male-male competition for access to females and mating opportunities (Bovero et al., 2003; Marvin, 2009). Pasmans et al. (2012) reported that male *Pa. shangchengensis*, with more developed masseter muscles, have a wider head than females, which is consistent with our result. Developed masseter muscles increase biting strength and increase male-male competitive ability. Thus, SD of head size in *Pa. shangchengensis* may contribute to sexual selection (male-male competition). Male biased of intercanthal space may related the development of muscles levator mandible anterior in males, because they are more enlargement in males (Fig. 1B), which also can increase male-male competitive ability.

Sexual dimorphism in trunk length (in this study, trunk length points to the distance between limbs) of salamanders is correlated to a fecundity advantage (Romano, Bruni & Paoletti, 2009). Females are usually longer than males (e.g., Marvin, 2009; Romano, Bruni & Paoletti, 2009; Zhang et al., 2014; Xiong et al., 2016a; Xiong et al., 2016b; Xiong et al., 2017). A longer trunk means a larger abdominal volume, which can produce larger clutches and/or a larger number of eggs (Shine, 1988); thus increasing reproductive capacity (Jockusch, 1997; Marvin, 2009). Like in most salamanders, female *Pa. shangchengensis* have a longer trunk than males, which may be the result of a fecundity advantage.

Two type of fertilization modes have been described in urodeles. External fertilization occurs in members of the families Hynobiidae, Crytobranchidae, and Sirenidae, whereas internal fertilization occurs in all other salamanders (Duellman & Trueb, 1994; Wells, 2007). Although SD of the limbs in salamanders (males usually have longer and more robust limbs) is associated with reproduction and competition (e.g., Malmgren & Thollesson, 1999; Seglie, Roy & Giacoma, 2010; Zhang et al., 2014; Reinhard, Renner & Kupfer, 2015), the limbs of salamanders with different fertilization modes have different functions. The males of most species of internally fertilized salamanders use their limbs (mainly forelimbs) to grip the females during amplexus (Duellman & Trueb, 1994). Thus, the longer and more robust limbs of males are better equipped to retain their grip on females and to resist a take-over by a competing male (Howard & Kluge, 1985), which benefits courtship performance (Rehak, 1983; Malmgren & Thollesson, 1999). The males of most species of externally fertilized salamanders use their limbs to grasp females, hold and embrace the egg sacs, and prevent fertilization interference by other males (e.g., Hasumi, 1994; Hasumi, 2015; Park, Park & Yang, 1996; Park & Park, 2000; Guo, Mi & Deng, 2008). Males with longer and more robust limbs maybe better equipped to increase their reproductive and competitive abilities. In this study, male *Pa. shangchengensis* had longer and more robust limbs than females. This dimorphic trait is also found in two hynobiid salamanders, e.g., *L. shihi* (Zhang et al., 2014) and *H. leechii* (Xiong et al., 2017). However, the reproductive biology of *Pa. shangchengensis* has not been reported, further studies are needed to be carried out in this facet, and test whether reproductive and competitive behaviors can explain the dimorphism of limbs.

Sexual dimorphism of tail (tail length and width of males are longer and wider than in females) was detected in *Pa. shangchengensis*, which was also found in other hynobiid salamanders, e.g., *O. zhangyapingi* (Xiong et al., 2016b), *H. kimurae* (Kakegawa et al., 2017), and *S. keyserlingii* (Hasumi, 2010). Dimorphism of tail length may attribute to energy storage (Xiong et al., 2016b; Kakegawa et al., 2017), which is one of the functions of tail (Bakkegard & Rhea, 2012). In this study, tail width is equal to the width of cloaca. Thus, dimorphism of tail width is equal to the dimorphism of cloacal width. In urodeles, males often have a more swollen cloaca (e.g., Kupfer, 2007; Reinhard & Kupfer, 2015) because of the hypertrophy of cloacal glands which secreting courtship pheromones play a major role in salamander mating and direct effect on male reproductive success (Reinhard & Kupfer, 2015). Thus male-biased tail width is benefits male reproductive success.

In amphibians, the formation of SD is not only relate to the drive forces (sexual selection, fecundity selection, and ecological selection), but to the age of animals. For example, sexual size dimorphism of *Tylototriton verrucosus* is result from the difference in the mean age at maturity (Seglie, Roy & Giacoma, 2010), head size dimorphism in *Aneides flavipunctatus* is a result of a higher head growth rate in males at sexual maturity relative to females (Staub, 2016). Thus, the effect of age should be account for the formation of SD. However, the research on the age of *Pa. shangchengensis* has not been carried out, future research should be carried out to determine whether the age is the source of SD in this salamander.

## Conclusion

In conclusion, *Pa. shangchengensis* used in this study showed obvious SD, including body size and shape SD. Sexual dimorphism of SSD, head, tail, and limbs toward male-biased, and trunk toward female-biased. Sexual dimorphism in body size and shape results from different selection forces or an equilibration of different selection forces. Although existing theories try to explain sexual size and shape dimorphism, future studies about the biology of *Pa. shangchengensis* should be carried out to confirm the explanations.

##  Supplemental Information

10.7717/peerj.6408/supp-1 Supplemental Information 1 The raw data of *Pachyhynobius shangchengensis*

## Additional Information and Declarations

### Data Availability

The following information was supplied regarding data availability: The raw measurements are available in the Supplemental File.
